# Identification of a novel RUNX2 gene mutation and early diagnosis of CCD in a cleidocranial dysplasia suspected Iranian family

**DOI:** 10.1002/ccr3.2825

**Published:** 2020-04-03

**Authors:** Omid Daneshjoo, Pirooz Ebrahimi, Leila B. Salehi, Antonio Pizzuti, Masoud Garshasbi

**Affiliations:** ^1^ Medical Genetics Group Department of Experimental Medicine “Sapienza’’ University of Rome Rome Italy; ^2^ Medical Genetics Department DeNA Laboratory Tehran Iran; ^3^ U.O.C. of Medical Genetics Policlinic of Tor Vergata Rome Italy; ^4^ Universal Scientific Education and Research Network Tehran Iran; ^5^ Department of Medical Genetics Faculty of Medical Sciences Tarbiat Modares University Tehran Iran

**Keywords:** autosomal dominant, cleidocranial dysplasia, *RUNX2* gene, sequencing

## Abstract

This research resulted in the identification and submission of a novel RUNX2 gene mutation in the affected members of the studied pedigree. Mutation screening is an effective method for the early diagnosis of CCD in the affected individuals.

## Graphical abstract

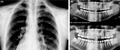

## 1 INTRODUCTION

Cleidocranial dysplasia (CCD) is an autosomal dominant (AD) skeletal dysplasia. The cause of CCD affection is usually a defect in the *RUNX2* gene. In this study, we have screened the *RUNX2* gene in 7 members of an Iranian family for revealing the cause of illness.

Cleidocranial dysplasia (CCD) is an autosomal dominant (AD) skeletal dysplasia which is also known as Marie and Sainton disease.^1^, ^2^ This disease consists of the condition including defective skull ossification, open fontanels, clavicular hypoplasia, delayed ossification of the pelvis, the late eruption of permanent teeth, malformed dental roots, and supernumerary teeth.^3^, ^4^, ^5^, ^6^, ^7^, ^8^ Although the intelligence of CCD affected individuals is normal, these patients suffer from their appearance characteristics related to the disturbance in the growth of the involved bones.^3^, ^9^, ^10^ The prevalence of CCD is one per one million approximately, which has been reported in different ethnic groups, and there is no gender differentiation among the CCD patients to the affection of this disease.^11^

Many cases are misdiagnosed because of the extreme variability of the skeletal and extraosseous symptoms. There are many difficulties in the definition of the particular criteria and the early diagnosis of CCD because the majority of the craniofacial abnormities becomes obvious only during adolescence, but this is confirmed the mutations in the *RUNX2* gene cause cleidocranial dysplasia.^12^, ^13^ The most striking marker, the extreme shoulder mobility, is not always expressed. Dentition anomalies occur in 93.5% of the CCD cases.^12^ Early recognition of the disorder is advantageous for successful therapy and rehabilitation. Follow‐up in CCD cases consists of skeletal and dental treatment procedures. When the jaws are fully developed, implant insertion and bridges are the final steps of the orthodontic therapy.^12^ The skeletal deficiencies treatment needs specialized orthopedic and physiotherapy activities.^14^

The cause of CCD affection is the presence of a defect in the *RUNX2* gene due to the haploinsufficiency of the gene product.^15^ The *RUNX2* gene is a member of the RUNX transcription factors family and is mapped to chromosome 6p21.1. This gene encodes a nuclear protein (Runt‐related Transcription Factor 1 or RUNX2 Protein) with a Runt DNA‐binding domain. This protein is essential for the regulatory factors that cause osteoblast differentiation from stem cells.^16^ RUNX2 protein also plays a fundamental role in skeletal gene expression, skeletal morphogenesis, osteoblast maturation‐homeostasis, and normal bone development.^9^, ^10^, ^15^ A study on the mice models with the *RUNX2* gene‐targeted homozygous disruption showed the absence of osteoblast differentiation with a complete lack of bone formation and change in early dental development. The mentioned study indicates the *RUNX2* gene is involved in fetal bone growth and dental tissue formation.^10^ This characterization is owing to the p21*^CIP/WAF1^* promoter expression by RUNX2 protein in fibroblasts and osteoblast lineage cells.^17^ Although a wide variation in the clinical features by this disorder has seen, CCD affected patients are known for exhibiting short stature, delayed closure of the cranial fontanels, frontal bossing, rudimentary or absent clavicles, and dental abnormalities.^7^, ^11^, ^18^, ^19^, ^20^ By the retention of primary dentition, delayed eruption and consequent impaction of the permanent teeth are a widespread phenomenon among the affected patients.^21^ Usually, the mentioned situation causes the presence of the multiple supernumerary teeth which requires long‐term orthodontic and complex dental treatment.^22^, ^23^

In this study, we have screened the *RUNX2* gene in 7 members of an Iranian family for revealing the defect in this gene that caused the illness. Suspicious affected individuals in this study had a disturbance in the normal clavicle bones growth associated with the short stature, malformed dental roots, and the late eruption of permanent teeth. This research resulted in the identification and submission of a novel *RUNX2* gene mutation in the affected members of the studied family (ClinVar Submission‐ID: SUB2986596). The significance of this case report is considering the importance of early diagnosis in the CCD curation procedure, mentioning the revealed mutation in Iranian ethnicity as a cause of CCD affection and showing the effectiveness of mutation screening in the *RUNX2* gene for the screening the affected individuals and early diagnosis.

## 2 CASE PRESENTATION

**Figure 1:**
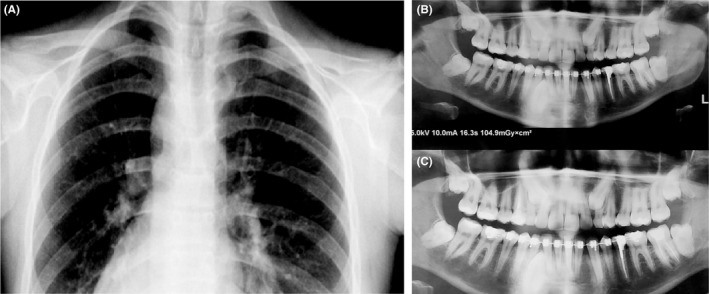
The radiograph images of bone clavicles and teeth in the member II:1. Hypoplastic form and incomplete growth of clavicle bones (A). Supernumerary teeth and the late eruption of permanent teeth (B, C). The patient was under orthodontic therapy at the time

**Figure 2:**
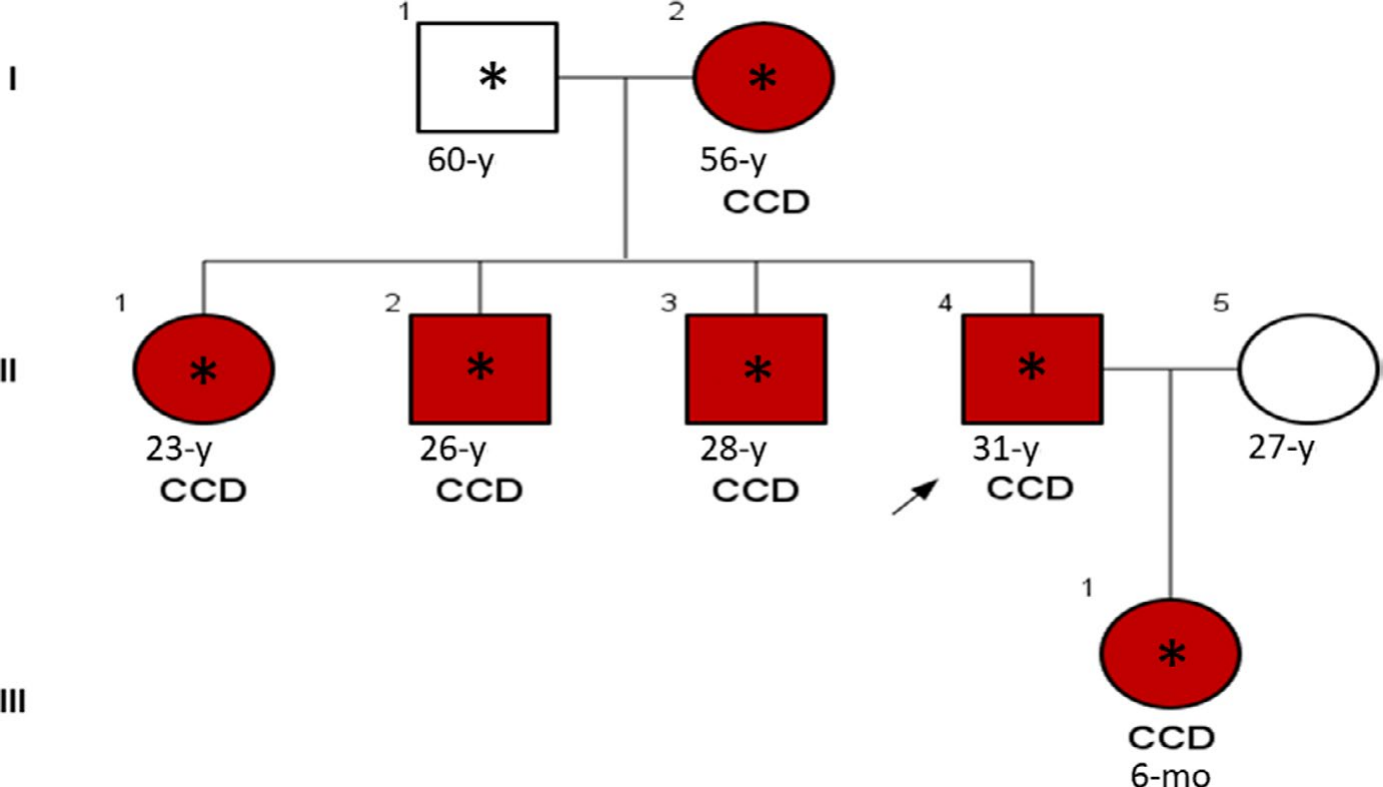
Pedigree of the Iranian family with Cleidocranial dysplasia. Red boxes denote affected individuals. Boxes with (*) indicate individuals that were analyzed by Sanger sequencing

The studied pedigree consisted of 6 affected members with similar clinical features involving the disturbance of the clavicle bones growth associated with the short stature, malformed dental roots, and the late eruption of permanent teeth. Figure 1 shows the clinical situation of the clavicle bones and teeth in the one affected member (member II‐1) in the pedigree. Figure 2 shows the pedigree of the studied family. All the affected individuals were alive at the time of this report. Table 1 indicates the clinical and molecular features of CCD patients in this study.

**Table 1:** The clinical and molecular features of CCD patients in this study

Patient ID	Gender	Age	Stature length (cm)	Clavicles formation	Number of supernumerary teeth	Delayed eruption of permanent teeth	Mutation
I:2	Female	56‐Y	148	Hypoplastic	5	+	c.860‐2A > G
II:1	Female	23‐Y	159	Hypoplastic	4	+	c.860‐2A > G
II:2	Male	26‐Y	165	Hypoplastic	3	+	c.860‐2A > G
II:3	Male	28‐Y	163	Hypoplastic	4	+	c.860‐2A > G
II:4	Male	31‐Y	168	Hypoplastic	4	+	c.860‐2A > G
III:1	Female	6‐M	Incomplete Puberty	Incomplete Puberty	Incomplete Puberty	Incomplete Puberty	c.860‐2A > G

All participants, or their legal guardian, were provided written and informed consent. The institutional review boards of Tarbiat Modares and the Sapienza University of Rome have reviewed the project. The affected individuals were under an examination at the Medical Genetics Department, DeNA laboratory, Tehran, Iran. Patients showed clinical features in similar severities.

DNA was extracted from peripheral blood of the patients I:2, II:1, II:2, II:3, II:4, III:1, and one healthy family member (I:1), using standard protocols.^24^

Table 2 depicts the sequence of primers used for amplifying the whole coding region and exon‐intron boundaries of the *RUNX2* gene. The primers were designed using Primer 3 software (version 0.4.0).^25^ The primers specificity was checked by in silico‐PCR and blat tools of the UCSC genome browser. Lack of SNPs in the genomic region corresponding to the 3' ends of the primers was checked by checking through the dbSNP database.

**Table 2:** Primer sequences used for PCR amplification of all exons and exon‐intron boundaries of the *RUNX2* gene

Exon1	F5′‐ tctgcctctccagtaatagtgc‐3′
	R5′‐ ctagcagtttatcaaagaatcatacct‐3′
Exon2	F5′‐ cttgcccctcatttccac‐3′
	R5′‐ aaggcaggaggtcttggag‐3′
Exon3	F5′‐ cattcctgtcggccattact‐3′
	R5′‐ tcagaaaaagaaggtgctgattt‐3′
Exon4	F5′‐ tcggagggtttccaatttag‐3′
	R5′‐ tgcagatagcaaagtccacaa‐3′
Exon5	F5′‐ ttgaaatggaaggcattatgtaga‐3′
	R5′‐ tccaggagttttgaagtgaaca‐3′
Exon6	F5′‐ ttttctctccctgtttttctgc‐3′
	R5′‐ ttgcccacatgcctctaata‐3′
Exon7	F5′‐ agggatgggaacctctctgt‐3′
	R5′‐ agggttaagtgccatgatgtg‐3′
First Part of Exon8	F5′‐ aggtctgtctgtggcttgct‐3′
	R5′‐ tttaatagcgtgctgccattc‐3′
Second Part of Exon8	F5′‐ ccaccactcactaccacacc‐3′
	R5′‐ ccctcttatggctgcaagat‐3′

PCR analysis was carried out in a total volume of 25 µL containing 0.5 µL of each forward and reverse primers (10 Pmol), 10 µL of PCR Master mix Mgcl2 1.5 mmol/L, and 1 µL DNA (about 100 ng). The reaction was adjusted to the total volume of 25 µL by ddH2O.^24^

The PCR was performed using an initial denaturation step at 94°C for 5 minutes, followed by 30 cycles of denaturation at 94°C for 30 seconds, annealing at 58 for 30 seconds, and elongation at 72°C for 30 seconds. PCR products were examined by 1% agarose gel electrophoresis for the presence and sizes of amplicons.^26^

Consequently, DNA sequencing of the PCR products was performed on 3130 ABI capillary electrophoresis using forward and reverse primers for each amplicon. The sequence chromatograms were analyzed by using CodonCode aligner software.^26^

In sample III:4, who was the proband of this family, sequencing of all exons and exon‐intron boundaries of the *RUNX2* gene revealed an important alteration on position chr6:45512244 (GRCh38.p7), : c.860‐2A > G.

The allele frequency of the identified variant in the *RUNX2* gene was determined by using the following public databases: dbSNP Common 144 (NCBI), 1K Genome project phase 3 (www.1000genomes.org), Exome Aggregation Consortium version 0.3 (ExAC), the Iranian Genome Project (https://irangenes.com/data‐2), and UK Biobank.

In silico pathogenicity prediction of the identified variant was performed using the following software: SIFT (http://sift.jcvi.org/), PolyPhen (http://genetics.bwh.harvard.edu/pph2/), Mutation Taster (http://www.mutationtaster.org/), and PROVEAN human genome variants (http://provean.jcvi.org/genome_submit_2.php). The prediction was that this variant is pathogenic and has damaging effects on the RUNX2 protein.

At last, conservation of the region harboring the variation was surveyed by comparing this region of the genome in the Human, Dog, Rhesus, Mouse, Elephant, Chicken, X‐tropicalis, Zebrafish, and Lamprey in UCSC database. The result of multiple alignments showed this region is highly conserved arguing that this locus plays an important role in the final structure formation and function of RUNX2 protein.

**Figure 3:**
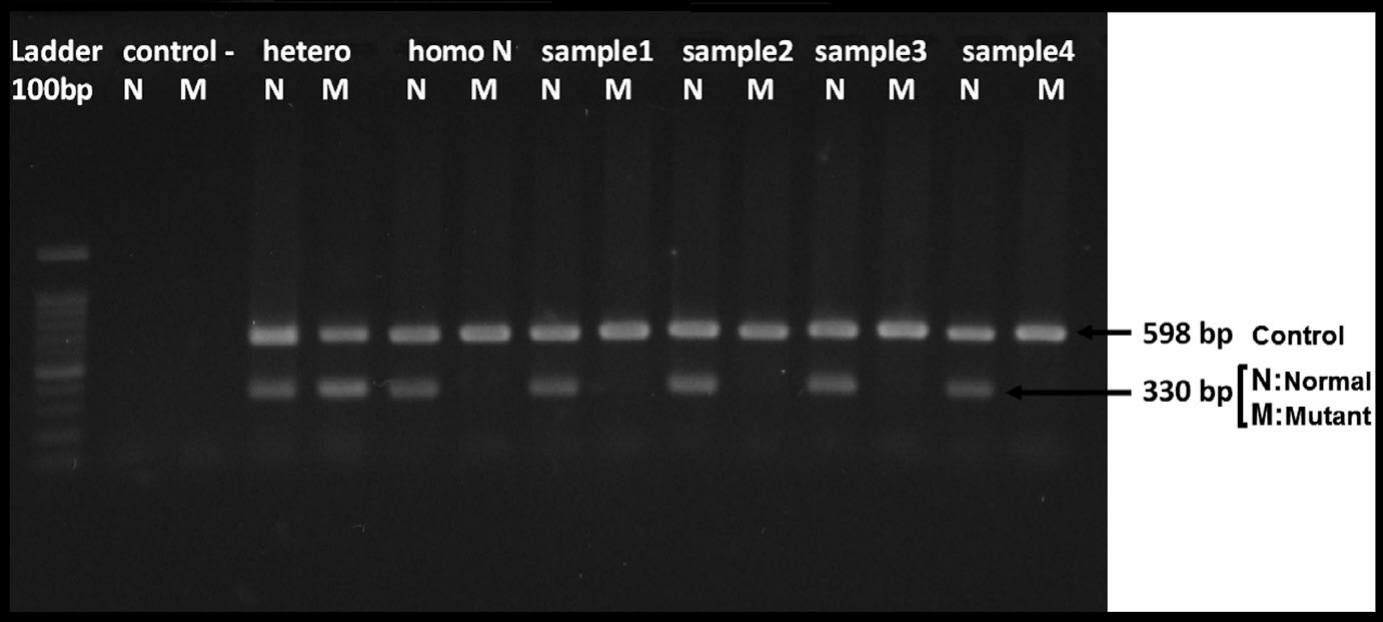
Picture of 2% agarose gel electrophoresis of ARMS‐PCR test products for variant c.860‐2A > G. Well 1:100 kb ladder. Well 2 and 3 (control‐): negative polymerase chain reaction test control (no template control; NTC). The Well 4 and 5 (Hetero): one affected individual with heterozygote state (proband) with a band in normal lane (N) and mutant lane (M). Well 6 and 7 (Homo N): one healthy individual with homozygote state and with a band just in the normal lane (N). Well 8‐15:4 samples of 100 Iranian ethnicity‐matched healthy controls. 598 bp Control band: one regular band with primers that are related to the sequence close to the *RUNX2* gene sequence. 330 bp normal band: the band which is related to designed primers for the normal allele. 330 bp mutant band: the band which is related to designed primers for the mutant allele

Standard ARMS‐PCR was employed to check the allele frequency of the mentioned variant in 100 healthy ethnicity‐matched controls. The controls were checked for : c.860‐2A > G variant in intron5 using the ARMS‐PCR method, and the result was negative for all 100 individuals (Figure 3). The primers and size of expected bands of each reaction are depicted in Table 3.^27^

**Table 3:** ARMS primer sequences used for checking c.860‐2A > G variant in healthy populations controls

Mutation	Primer	Sequence	The expected size of product fraction
c.860‐2A > G	Mutant allele primer (Forward)	TACTAAAGATTTTTCTTTTTCTTTTTCCAG	330 bp
	Normal allele primer (Forward)	TACTAAAGATTTTTCTTTTTCTTTTTCCAA	
	Reverse primer	ATTTGCCAGTTGTCATTCCC	
	Control primer (Forward)	TGCTCATTCTCTTTTTGTTTTG	598 bp
	Control primer (Reverse)	TTTCTTCTGGTGAGGGTTAATG	

**Figure 4:**
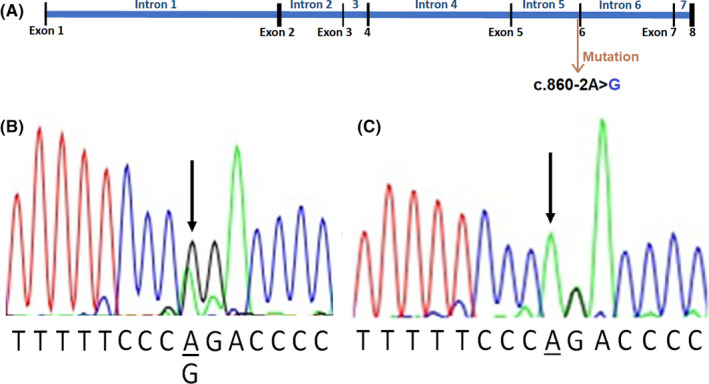
Schematic presentation of *RUNX2* gene structure and c,860‐2A > G position (A), Sanger sequencing peaks which show the c.860‐2A > G mutation in intron5 of the *RUNX2* gene in all affected members (B), a normal variant of this region in the unaffected member (C)

In sample III:4 who was the proband of this family sequencing of all exons and exon‐intron boundaries of the *RUNX2* gene (Figure 4A, Table 2) revealed >: c.860‐2A > G alteration at the chr6:45512244 position (GRCh38.p7). The mentioned alteration was present in all other affected members of this pedigree also (Figure 4B), and all unaffected members showed the normal variant (c.860‐2A) in this position (Figure 4C).

The allele frequency of c.860‐2A > G was less than 0.01 in NCBI, 1000 Genomes Project, ExAC, the Iranian Genome Project, and UK Biobank databases.

The c.860‐2A > G variant is the second prior base to the exon6 initiator base in the *RUNX2* gene at the position chr6: 45512244 (GRCh38.p7). This variant is a point mutation at the Intron5‐Exon6 splice site boundary (5′**A**GC3′‐>5′**G**GC3′) which causes a disturbance in the normal Intron5‐Exon6 splice site action.

**Figure 5:**
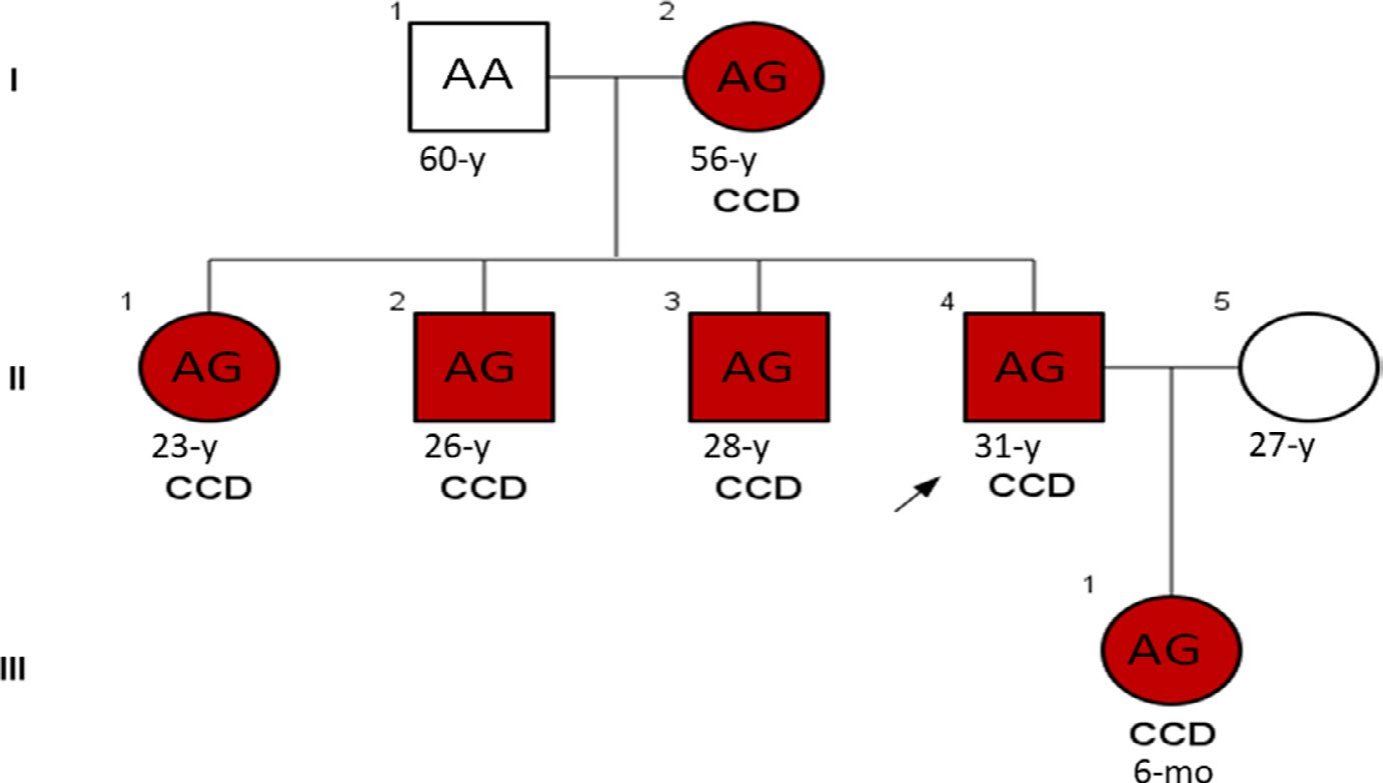
Segregation of the c.860‐2A > G variant in the *RUNX2* gene among the individuals of studied pedigree

The segregation analysis of this variant in the studied family was checked by Sanger sequencing in all available samples (Figure 5). The mentioned analysis indicated the : c.860‐2A > G mutation in the *RUNX2* gene is cosegregating with the disease in this family, and all affected individuals had the same mutation at chr6: 45512244 (GRCh38.p7) position.

All affected members were heterozygous for this mutation which confirmed the dominant Inheritance of this disease.

By the screening of the *RUNX2* gene for the proband's child (member IV:1), we figured out she received the mutant allele. The physiological evaluation for this person was not satisfying for diagnosis due to her age (6 month).

## 3 DISCUSSION

In this study, after clinical evaluation of the proband and the Sanger sequencing of his *RUNX2* gene, we were revealed a novel : c.860‐2A > G mutation in this gene. The segregation analysis of the involved pedigree confirmed the cosegregation of the mentioned mutation and its inheritance in the family. The clinical and physiological evaluation was not singly satisfying for a complete diagnosis of this disease, and the Sanger sequencing method was an effective assay for confirmation of the disease affection. Confirmation of CCD affection by mutation screening resulted in the early diagnosis, better follow‐up, and acceptable management of orthodontic difficulties in member II:1 (Figure 1B).

Various researches among different populations on this disease have been shown the variable phenotypes in the affected individuals, even in the one family with the same mutation.^28^ It seems there is no clear correlation between the type of mutation and the phenotype manifestation, as well as the correlation between the subpopulation origin and phenotype manifestation in the affected individuals.^19^, ^29^, ^30^

Regarding the identification of c.860‐2A > G mutation in the fifth intron 3′ splice site of the *RUNX2* gene, tow pathways are more possible to occur in the way of expression of the mutated gene.

**Figure 6:**
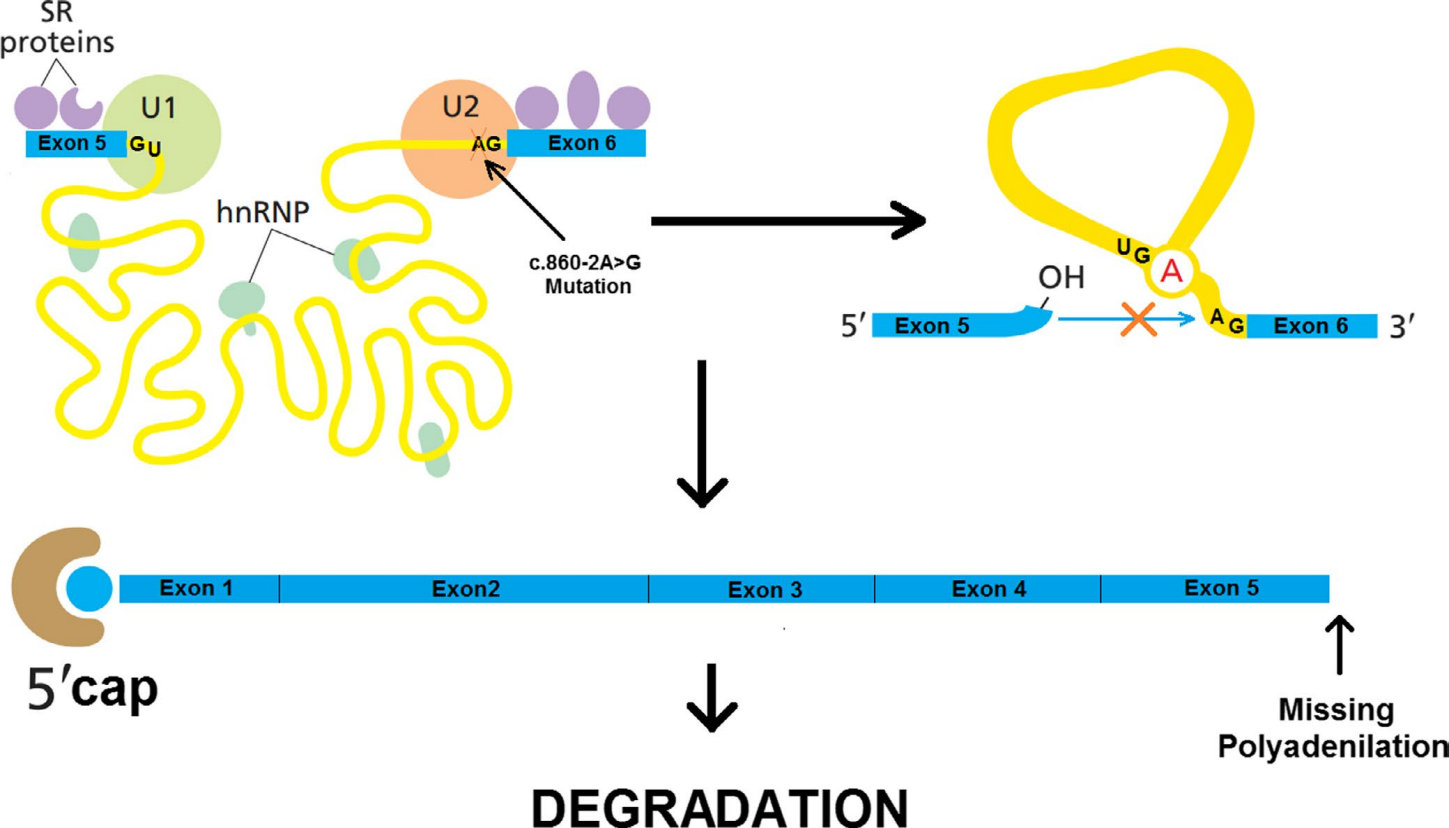
Schematic presentation of the defect in intron 5 splicing for the *RUNX2* pre‐mRNA

**Figure 7:**
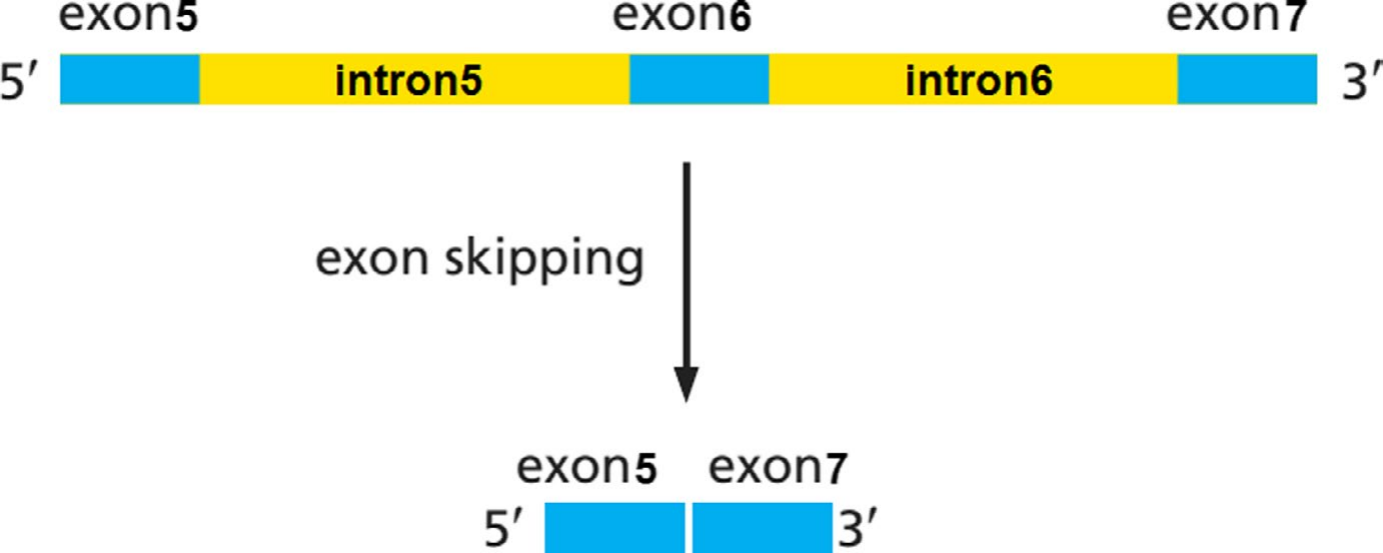
schematic presentation for skipping of exon6 in *RUNX2* mRNA

- Due to mutation in 3′ splice site of the fifth intron in the *RUNX2* transcript, splicing after exon5 could face disruption and causes truncation of the mRNA product. Production of the mRNA with 5 exons (exons 1‐5) and without 3 remain exons (exons 6‐8) and polyadenylation at the end of it (Figure 6) could result in the mRNA instability and degradation of mentioned mRNA.^31^
- Mutated 3′ acceptor splice site (c.860‐2A > G) could act as a cryptic splice site and results in the elimination of exon6 in the transcription procedure (Figure 7).^32^

For clarification of the exact molecular happening and realizing the cause of deficiencies in the patient's skeletal, dental development pathways, further investigation on the RNA and Protein levels is required. One study on the CCD protein level indicated the variable loss of function of CCD protein due to alterations in the Runt and PST domains of this protein may give rise to clinical variability.^30^ The result of this research gives the motivation for further investigation toward the evaluation of mRNA‐Protein levels and variable loss of function of the mutant gene product.

## 4 CONCLUSION

Regarding witnesses, the mutated : c.860‐2A > G allele is the cause of CCD affection in the studied pedigree and this mutation has been inherited from member I:1 who was not in the access at the time of this research. The inheritance pattern of this disorder is autosomal dominant (AD) which is confirmed by the analysis of the mutation segregation in the pedigree. Mutation screening of the *RUNX2* gene in patients with CCD related features is an effective method for the early diagnosis of CCD in the affected individuals.

## CONFLICT OF INTEREST

In this study, the authors have no conflict of interest.

## AUTHORS’ CONTRIBUTIONS

MG: examined patients and collected the data. OM: wrote, analyzed the data, and reviewed this report. PE, L B.S, and AP: contributed to the interpretation of data. All authors: approved the final version of the manuscript.

## ETHICAL APPROVAL

All procedures were under the ethical standards of the national research committee.

## Data Availability

The result of genetic analysis is available on the ClinVar database (ClinVar Submission‐ID: SUB2986596).
